# Comparative transcriptome analysis of mammary epithelial cells at different stages of lactation reveals wide differences in gene expression and pathways regulating milk synthesis between Jersey and Kashmiri cattle

**DOI:** 10.1371/journal.pone.0211773

**Published:** 2019-02-05

**Authors:** Shakil Ahmad Bhat, Syed Mudasir Ahmad, Eveline M. Ibeagha-Awemu, Basharat A. Bhat, Mashooq Ahmad Dar, Peerzada Tajamul Mumtaz, Riaz A. Shah, Nazir A. Ganai

**Affiliations:** 1 Division of Animal Biotechnology, Faculty of Veterinary Sciences and Animal Husbandry, SKUAST-Kashmir, India; 2 Agriculture and Agri-Food Canada, Sherbrooke Research and Development Centre, Sherbrooke, Quebec, Canada; 3 Department of Life Science, Shiv Nadar University, Greater Noida, Uttar Pradesh, India; 4 Division of Animal Genetics and Breeding, Faculty of Veterinary Sciences and Animal Husbandry, SKUAST-Kashmir, India; University of Illinois, UNITED STATES

## Abstract

Jersey and Kashmiri cattle are important dairy breeds that contribute significantly to the total milk production of the Indian northern state of Jammu and Kashmir. The Kashmiri cattle germplasm has been extensively diluted through crossbreeding with Jersey cattle with the goal of enhancing its milk production ability. However, crossbred animals are prone to diseases resulting to unsustainable milk production. This study aimed to provide a comprehensive transcriptome profile of mammary gland epithelial cells at different stages of lactation and to find key differences in genes and pathways regulating milk traits between Jersey and Kashmiri cattle. Mammary epithelial cells (MEC) isolated from milk obtained from six lactating cows (three Jersey and three Kashmiri cattle) on day 15 (D15), D90 and D250 in milk, representing early, mid and late lactation, respectively were used. RNA isolated from MEC was subjected to next-generation RNA sequencing and bioinformatics processing. Casein and whey protein genes were found to be highly expressed throughout the lactation stages in both breeds. Largest differences in differentially expressed genes (DEG) were between D15 vs D90 (1,805 genes) in Kashmiri cattle and, D15 vs D250 (3,392 genes) in Jersey cattle. A total of 1,103, 1,356 and 1,397 genes were differentially expressed between Kashmiri and Jersey cattle on D15, D90 and D250, respectively. Antioxidant genes like RPLPO and RPS28 were highly expressed in Kashmiri cattle. Differentially expressed genes in both Kashmiri and Jersey were enriched for multicellular organismal process, receptor activity, catalytic activity, signal transducer activity, macromolecular complex and developmental process gene ontology terms. Whereas, biological regulation, endopeptidase activity and response to stimulus were enriched in Kashmiri cattle and, reproduction and immune system process were enriched in Jersey cattle. Most of the pathways responsible for regulation of milk production like JAK-STAT, p38 MAPK pathway, PI3 kinase pathway were enriched by DEG in Jersey cattle only. Although Kashmiri has poor milk production efficiency, the present study suggests possible physicochemical and antioxidant properties of Kashmiri cattle milk that needs to be further explored.

## Introduction

Mammary gland development and the physiological control of its dynamics are a vital part of the mammalian reproduction strategy [1–2]. Milk evolved as an essential source of nutrients and immune factors including immune-modulatory, anti-inflammatory and anti-microbial agents that offer protection against infections [3–4]. Milk yield and quality are important economic traits. An increase in the efficiency of milk synthesis both in terms of quality and quantity is a highly desirable goal for the dairy industry [5]. The mammary gland displays a high level of developmental plasticity with the ability to undergo repeated cycles of growth and regression [6]. Lactation is a dynamic physiological process characterized by an initial rapid increase in milk yield during early lactation, which peaks around 6 weeks into lactation, followed by a gradual decrease until the end of lactation [7]. The knowledge of gene expression involved in lactation informs on the biological mechanisms underlying mammary morphogenesis and metabolic activities as well as enhances our understanding of milk composition [8–9]. The ability to manipulate lactation performance in less improved breeds is an area of increasing interest, and knowledge of the biological pathways and mechanisms that govern mammary gland development and lactation may help to increase the lactation performance of dairy animals. Recent developments in “omics” technologies like transcriptomics make it possible to comprehensively and systematically identify the potential factors or processes that may influence lactation [10–11]. Using high throughput RNA sequencing technique, a high number of genes were identified as differentially expressed between different stages of lactation, and the expression alterations may play crucial roles in the regulation of lactation [9–12]. Thus, a thorough and deeper understanding of the genes and biological networks that regulate bovine milk composition is required.

Cow milk contains a heterogeneous population of somatic cells consisting of lymphocytes, neutrophils, macrophages and exfoliated epithelial cells [13]. Mammary epithelial cells (MEC) are unique in that they are involved in the synthesis and secretion of milk. Although milk somatic cells have been widely used to analyse the expression of genes involved in milk synthesis in ruminants [14–16], it is known that some milk trait genes of interest (e.g. genes in apoptosis pathway) are not solely expressed in MEC, but also by other cell types like leucocytes [17]. Thus, compared with MEC, there is the possibility to study genes not specifically expressed in MEC when milk somatic cells are used to study the expression of genes involved in milk synthesis. Moreover, Sciascia et al. [18] reported that milk somatic cells are not suitable for measuring milk protein expression in lactating ruminants. Although many studies have examined the physicochemical properties of cow milk and the genes expressed in the bovine mammary gland [19–22], limited research has detailed the characterization of genes expressed in milk epithelial cells. Therefore, identification and characterisation of milk quality and yield related genes expressed at different stages of lactation in MEC may represent an important step towards understanding of the complex biology of the milk production process.

The Jersey breed is amongst top milk producers in the world and it is routinely used to upgrade the milk producing capacity of the Kashmiri local cattle of North India. Kashmiri cattle are poor-performing and not improved for milk production, and differ greatly from Jersey in dairy production characteristics. Given the importance of the Kashmiri cattle in crossbreeding programs for augmenting milk production, this study aimed to compare its MEC transcriptome, using RNA Seq, with that of Jersey breed to gain a better understanding of the genes and pathways underlying the different milk producing abilities of the two breeds. We therefore report for the first time the MEC transcriptome of Kashmiri cattle at different stages of lactation using RNA-seq. We also present a characterization of the gene expression profile and differences between the MEC transcriptomes of Kashmiri and Jersey cattle.

## Materials and methods

### Animals and sampling

Animal care and use procedures were approved by the Institutional Animal Ethics Committee of Sher-e-Kashmir University of Agricultural Sciences and Technology of Kashmir. Three healthy Kashmiri and three Jersey cows in their 3^rd^ lactation at the Share-Kashmir University of Agricultural Sciences and Technology dairy farm, Mountain Livestock Research Institute (MLRI), Kashmir, India were selected for the study. The animals were kept in free stall housing, fed with balanced ration and had *ad libitum* access to water. Fresh milk samples (1.5 L) were aseptically collected by milking equally the four quarters of the cows on day 15 (D15), D90 and D250 in milk representing early lactation, mid-lactation and late lactation stages, respectively. Under the management conditions at the MLRI dairy farm, the lactation stages for Jersey cattle are D1-D80 (early lactation), D81-D185 (mid-lactation) and D186-D300 (late lactation). The corresponding periods for Kashmiri cattle are D1-70 (early lactation), D71 to D180 (mid-lactation) and D181-D280 (late lactation). Thus D15, D90 and D250 were chosen to represent early, mid and late lactation stages, respectively in both breeds. In total, nine samples per breed were collected. The milk samples were immediately transported to the laboratory in ice cooled containers. For milk quality analysis, the different parameters like milk yield/day, fat and protein content were recorded for ±7 days relative to day of sampling (i.e. seven days before day of sampling, day of sampling and seven days after day of sampling) for each lactation stage. The fat and protein contents were determined by Milk auto-analyser (Speedy Lab, Astori, Italy).

### Isolation of milk epithelial cells and purity check

Milk epithelial cells were isolated from whole fresh milk following the protocol of Boutinaud *et al*. [13] with some modifications. Milk sample (1.5 L/cow) was aliquoted (125 ml) into each of six 250 ml centrifuge tubes, and 100 ml cold (4°C) diethylpyrocarbonate (DEPC) treated 1 x phosphate buffered saline (PBS) buffer added. The samples were defatted by centrifugation for 20 min at 2800 x g at 4°C. The fat layer and whey portion were carefully removed and the remaining fraction (1 ml) at the bottom containing the cell pellet was mixed with 800 μl cold 1 x PBS and transferred into a 2 ml tube. After adding 200 μl EDTA (0.5 M pH 8.0, 4°C), the sample was centrifuged at 14,000 x g for 1 min at 4°C. The supernatant was discarded, and pellets of the same sample were pooled and resuspended in 200 μl cold 1 x PBS and centrifuged at 5100 x g for 5 min at 4°C. The supernatant was discarded and the pellet was resuspended in 1.25 ml cold 1 x PBS containing 1% bovine serum albumin (BSA, Sigma, USA). A portion (500 μl) of the resuspended milk somatic cells (MSC) was used for RNA isolation while the other portion was further purified to obtain MEC. Specific anti-cytokeratin peptide 18 antibody (KRT18, Clone KS-B17.2, Sigma–Aldrich, USA) coated beads (Dynabeads Pan Mouse IgG, Invitrogen, USA) were used to separate MEC from other cell types according to Boutinaud *et al*.,[13]. Briefly, 25 μl of Dynabeads was transferred to a 1.5 ml tube and washed twice with 1 ml 1% BSA–PBS to remove the preservative. The Dynabeads were resuspended in 1 ml 1% BSA–PBS and transferred to a 1.5 ml tube containing 3 μl of KRT18 antibodies. The suspension was incubated for 30 min at 4°C on a Sample Mixer (Rotospin, Tarson,-India). Then, the tube was placed in the magnetic particle concentrator (Dyna Mag 5, Invitrogen, USA) for 30 sec. After another wash step and aspiration of the supernatant containing unbound antibodies, the antibody-coated Dynabeads were resuspended in 250 μl 1% BSA–PBS and transferred to the 1.25 ml cell suspension and incubated for 1 h at 4°C on the Sample Mixer. Finally, specifically bound cells were collected by magnetic incubation for 1 min. The bead bound cell pellet was washed (1 ml 1 x PBS added followed by centrifugation at 4000g for 1 min) and immediately used for RNA extraction. Possible contamination of purified MEC was checked by quantification of the expression of marker genes for various MSC types like beta casein (*CSN2*, mammary epithelial cell marker), cytokeratin 18 (*KRT18*, epithelial cell marker), lymphocyte-specific protein one (*LSP1*, leucocyte specific cell marker), haemoglobin sub-unit alpha (*HBA*, red blood cell marker) and *CD18* (macrophage cell marker) [23–24] in samples collected on D90 by real time quantitative PCR (qPCR) (S1 Table).

### RNA extraction and sequencing

Total RNA extraction from MEC and MSC was accomplished by Trizol method (Ambion, USA) according to the manufacturer’s instructions. RNA was quantified by spectrophotometer (ThermoFisher, USA) and the quality and integrity was assessed by Bioanalyzer (Agilent, USA). The RNA integrity number (RIN) of samples ranged from 6.5–9.3. Only those samples having RIN values above 8 were used for library construction. The RNA isolation process was repeated for samples with lower RIN values until a RIN value of ≥8 was achieved.

Illumina TruSeq stranded mRNA sample preparation kit was used to generate cDNA libraries according to the manufacturer’s recommendations. Total RNA (4μg/sample) was used to prepare the libraries. Poly-A containing mRNA molecules were purified using poly-T oligo-attached magnetic beads. Following purification, RNA was fragmented into small pieces of 300 bp size using divalent cations under elevated temperature. The cleaved RNA fragments was used to synthesize first strand cDNA using reverse transcriptase and random primers (Illumina, USA) followed by second strand cDNA synthesis using DNA Polymerase I and RNase H. After adenylation of 3’ ends of DNA fragments, hybridisation was initiated by ligating Illumina paired-end adapter and index. cDNA fragments (200bp) were generated and were selectively enriched to construct the final sequencing paired end library using Illumina PCR Primer Cocktail. Libraries were pooled in equimolar amounts and paired end sequenced (126 bp) on three lanes (6/lane) on a High Throughput Model flow cell on an Illumina HiSeq 2500 platform by SciGenom, Cochin, Kerela-India.

### Sequence data processing, alignment and identification of expressed genes

Raw data (reads) in fastq format were first processed by removing adapter sequences and reads having a phred score <30 with Trimmomatic software v0.32. Clean reads were aligned to the bovine reference genome, UMD3.1 version 85 (ftp://ftp.ensembl.org/pub/release-85/gtf/bos_taurus/Bos_taurus.UMD3.1.85.gtf.gz) with Bowtie v2.0.6. Also, a data-base of splice junctions was generated by TopHat v2.1.1[25–26] (http://tophat.cbcb.umd.edu/) based on gene model annotation file 77 (ftp://ftp.ensembl.org/pub/release-77/gtf/bos_taurus).

### Differential gene expression analysis

Aligned reads were assembled with Cufflinks and differentially expressed genes (DEG) between lactation stages and breed were detected and quantified with Cuffdiff [26]. Negative binomial distribution was used to calculate gene expression which was normalized in fragments per kilobase of transcript per million mapped reads (FPKM). T-test was used to identify significantly differentially expressed genes and gene expression differences were declared significant at p-values < 0.05 after Benjamini-Hochberg correction.

### Gene ontology (GO) and KEGG analysis of differentially expressed genes

GO and pathway enrichment analysis of DEG was accomplished with Gene Ontology Consortium data base (http://www.geneontology.org) [27]. GO terms and KEGG pathways (http://www.genome.jp/kegg/) with Benjamini and Hochberg corrected p-values < 0.05 were considered significantly enriched.

### Protein-protein interaction networks of differentially expressed genes

Protein-protein interaction (PPI) networks were constructed on the basis of information from STRING v10.5 (https://string-db.org), using the Ensemble gene identifiers of DEG as input and *Bos taurus* as background which provides critical assessment and integration of protein-protein interactions, including direct (physical) and indirect (functional) associations [28]. The top 20 DEG and DEG for milk traits from lactation stage comparisons in Kashmiri and Jersey cattle were used in protein-protein interaction analysis. Credible interactions (combined_score ≥ 0.4) were further visualized using CytoScape [29].

### Quantitative real time PCR

Real time quantitative PCR was performed to verify the expression levels of eight DEG (*GPAM*, *BDH1*, *SLC2A1*, *SLC2A8*, *HK2*, *SOS2*, *FAS* and *XDH*) involved in different milk synthesis pathways. cDNA was synthesized from 0.5 μg of the same total RNA used in RNA sequencing using the Revert Aid First Strand cDNA Synthesis Kit (Thermo Scientific, USA) as per the manufacturer’s protocol. Primers were designed using Primer3 Plus software (https://primer3plus.com/cgi-bin/dev/primer3plus.cgi). qPCR was performed on a Light Cycler 480 II Real-Time PCR System (Roche, Switzerland). The reaction volume of 20 μl included 10 μL of 2X SYBR Green MasterMix reagent (Thermo Scientific, USA), 1μl of cDNA and 0.2 μL of each primer (10μM). The sequences of the primers and annealing temperatures are shown in S1 Table. All reactions were conducted in triplicates and included negative controls with no template. The expression levels of genes were normalized with *GAPDH* and *UXT*. *GAPDH and UXT* were initially tested and shown to be stable under the experimental conditions. The relative gene expression was calculated using the 2^-ΔΔCt^ method [30].

## Results

### Milk yield traits

Milk yield (kg/day), fat and protein contents were determined for ±7 days relative to day of sampling for each lactation stage. The milk yield per day varied significantly between lactation stages in both Kashmiri and Jersey cattle. The mid lactation was characterised by highest milk yield (p<0.05), whereas protein and fat contents were maximum (p<0.05) during initial stages of lactation in both breeds, except that early lactation protein content (3.21±0.58) was similar to late lactation protein content (3.11±0.39) in Kashmiri cattle (Table 1). As expected, the milk yield of Jersey cattle was higher than that of Kashmiri cattle at all lactation stages. The fat and protein contents in Jersey cattle ranged from 4.10% to 4.85% and 2.91% to 3.36%, respectively. The corresponding values for Kashmiri cattle were 3.20%-3.94% and 2.81%-3.21%, respectively.

**Table 1:** Milk yield and component traits^1^ in Kashmiri and Jersey cattle at different stages of lactation.

Lactation stage	Jersey cattle			Kashmiri cattle		
	Milk yield (kg/day)	Protein (%)	Fat (%)	Milk yield (kg/day)	Protein (%)	Fat (%)
Early lactation	8.2±0.95^a^	3.36±0.72^a^	4.85±0.93^a^	4.12±0.89^d^	3.21±0.58^d^	3.94±0.62^d^
Mid lactation	10.5±1.1^b^	2.91±0.36^b^	4.10±0.78^b^	5.20±0.99^e^	2.81±0.49^e^	3.20±0.77^e^
Late lactation	6±0.81^c^	3.21±0.78^b^	4.63±0.62^c^	3.82±0.62^f^	3.11±0.39^d^	3.46±0.99^e^

^1^Values are the means ± standard deviation of data collected ±7 days relative to day of sampling at each lactation stage (D15, D90 and D250).

^a, b, c, d, e^For each parameter and breed, column means with different superscripts differ significantly.

### Sequencing and expressed genes in mammary epithelial cells

**Fig 1:**
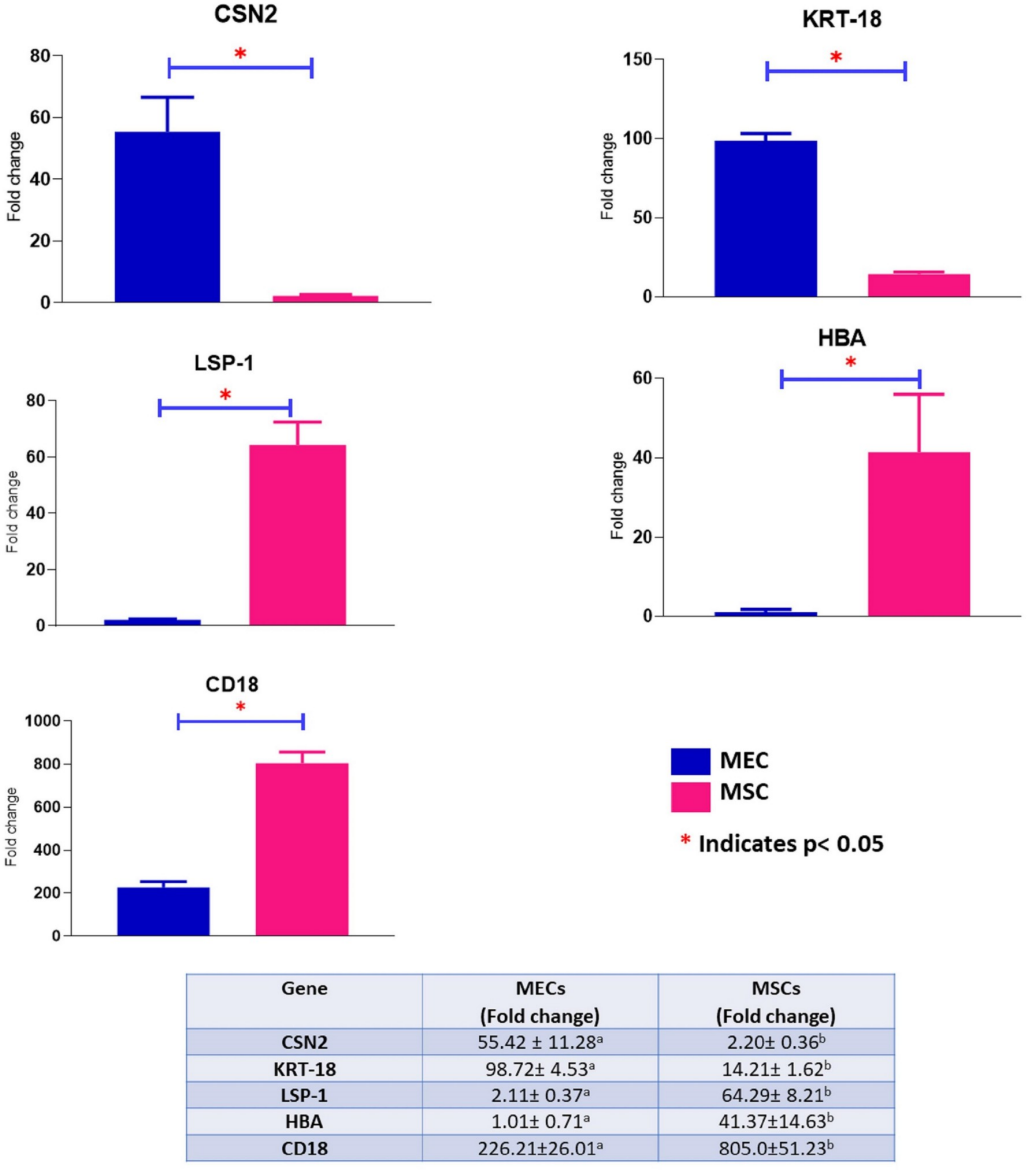
Comparison of the expression levels of different cell type marker genes in isolated mammary epithelial cells (MEC) as compared to milk somatic cells (MSC).

To test the purity of isolated MEC and validate its use in transcriptional studies of milk trait genes, the expression of marker genes for specific cell types was compared between MEC and MSC. The expression levels of marker genes were normalised against *GAPDH* and *UXT* housekeeping genes. The mRNA expression levels of *KRT18* and *CSN2* were significantly higher (p<0.05) in the isolated MEC as compared to MSC (Fig 1). Furthermore, the expression of *LSP1*, *HBA* and *CD18*, chosen as markers for cell types other than MEC, were significantly higher (p<0.05) in MSC as compared to MEC (Fig 1).

Sequencing of 18 libraries generated a total of 1.65 billion reads (range 68,43–136,83 million reads/library) (S2 Table). Out of this number, 1.47 billion reads (95.82%) passed quality control and were aligned to the bovine genome UMD3.1. A total of 1.44 billion uniquely mapped reads were further processed while reads that mapped to multiple positions, unaligned and discordant reads were discarded (S2 Table). Mapped genes with FPKM ≤0.01 were discarded and the remainder divided into a low expression group (< 10 FPKM), a moderate expression group (10 FPKM to 500 FPKM) and a high expression group (> 500 FPKM) (S3 Table). For both breeds, the highest number of expressed genes with FPKM >0.01 was during late lactation (D250) in Jersey (13,835 genes) and Kashmiri cattle (14,464 genes) (S3 Table).

### Top most expressed genes at each stage of lactation in Kashmiri and Jersey cattle

The numbers of genes with the highest FPKM values (> 500) in each breed and lactation day are shown in S3 Table. For both breeds, 13 top expressed genes at each lactation day accounted for ~70% of the total FPKM values (Table 2). The top expressed genes at each stage of lactation were similar for both breeds, except *RPS12* and *CCL14*, which were highly expressed in Kashmiri cattle only or Jersey cattle only, respectively.

**Table 2:** Highly expressed genes in mammary epithelial cells with FPKM values >2000 at three different stages of lactation in Kashmiri and Jersey cattle^1^.

Genes	Kashmiri			Jersey		
	D15	D90	D250	D15	D90	D250
CSN1S1	151,546	128,456.2	194,567.1	164,564.5	156,743.9	234,598.2
CSN1S2	71,398.8	89,764.6	125,657	150,945	167,512.3	182,657.2
CSN3	161,987.2	159,574.6	192,456.5	181,457.6	150,675.1	231,241.7
CSN2	211,651.8	185,657.23	227,861.2	243,561.9	200,165	245,241.9
LGB	147,876.25	98,365.7	148,641.1	137,645.7	100,387	140,034.5
LALBA	34,224.31	37,937.7	52,945.3	41,669.1	40,597.8	45,782.2
RPLP1	21,400.1	15,712.7	7,041	9,184.13	3,006.93	3,774.83
RPS28	17,835.6	9,313.4	3,436.38	8,888.89	-	-
RPS20/snoU54	14,922.6	6,510.56	-	8,756.15	-	-
RPLPO	11,925.8	-	3,133.07	5,337.15	-	-
RPS12	-	-	-	-	3,089.68	2,494.05
B2M	-	7,468.17	2,723.17	-	10,721	14,357.5
CCL14	-	-	-	-	7,509.43	10,501.5

^1^Mammary epithelia cells were isolated from milk obtained from Kashmiri (n = 3) and Jersey (n = 3) cows at D15 (15 days in milk) (early lactation), D90 (mid-lactation) and D250 (late-lactation) and subjected to RNA-sequencing.

‘-’Indicate that the genes have FPKM values less than the threshold values (See S3 Table for FPKM values of all genes).

### Differentially expressed genes between lactation stages in Kashmiri and Jersey cattle

**Fig 2:**
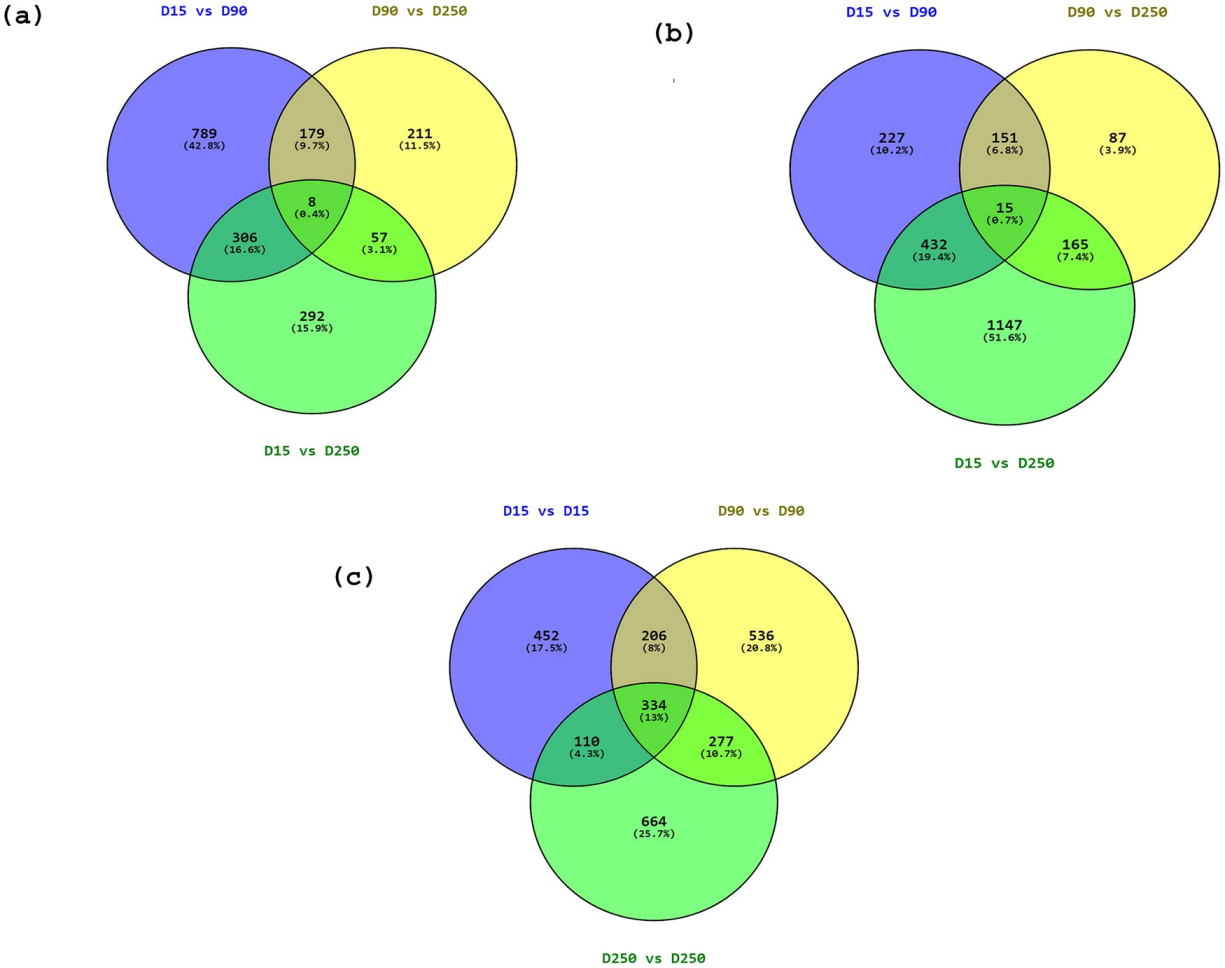
Significantly differentially expressed genes between D15 vs D90, D15 vs D250 and D90 vs D250 in mammary epithelial cells in (a) Kashmiri and (b) Jersey cattle and (c) D15, D90 and D250 between Jersey and Kashmiri cattle.

A total of 1,282, 455 and 665 genes were differentially expressed (FDR<0.05) between D15 vs D90, D90 vs D250, and D15 vs D250, respectively in Kashmiri cattle (Fig 2a, S4 Table). Likewise, 826, 418 and 1,765 genes were differentially expressed (FDR<0.05) between D15 vs D90, D90 vs D250, and D15 vs D250, respectively in Jersey cattle (Fig 2b, S4 Table). The largest number of DEG were observed between D15 vs D90 in Kashmiri (1,282 genes) and between D15 vs D250 in Jersey (1,765 genes). The number of DEG that were common to all lactation stages were 8 and 15 in Kashmiri and Jersey, respectively. The top ten DEG with highest fold changes for each breed are listed in Table 3.

**Table 3:** Top 10 differentially expressed genes in mammary epithelial cells with highest fold changes between lactation stages in Kashmiri and Jersey cattle^1^.

Gene	D15 vs D90			D90 vs D250				D15 vs D250			
	Log2Fold change	p-value	FDR^2^	Gene	Log2Fold change	p-value	FDR	Gene	Log2Fold change	p-value	FDR
**Kashmiri cattle**											
SNORD50	-11.066	5E-05	0.001	B3GNT6	7.031	0.001	0.010	PEAR1	7.008	0.003	0.031
TMEM232	-11.216	5E-05	0.001	PEAR1	5.771	0.0003	0.006	DMP1	6.936	0.001	0.018
ATP6V0D2	6.038	0.005	0.044	TMEM232	5.117	0.003	0.032	SLC18B1	6.768	0.003	0.0288
CPM	5.144	0.0001	0.002	LMO7	5.106	5.0E-05	0.001	SNORD50	-12.055	5E-05	0.0013
SPDEF	-6.378	5E-05	0.001	ME1	4.856	0.004	0.038	TMEM232	-10.875	0.001	0.016
FHOD3	-5.217	0.001	0.019	PLEKHF1	-4.857	5.0E-05	0.001	MAP2	6.291	5.0E-05	0.001
MROH2B	-5.151	0.0002	0.004	F3	4.846	5.0E-05	0.001	B4GALT6	6.193	0.0009	0.013
INSC	-4.979	5E-05	0.001	DCN	4.787	0.001	0.020	ESR1	6.072	0.0005	0.008
HACD4	4.905	0.0004	0.006	CLDN1	4.772	5.0E-05	0.001	CLDN1	6.038	0.001	0.020
LRRC66	4.886	0.002	0.028	HSPB8	4.530	5.0E-05	0.001	CPM	5.924	5.0E-05	0.001
**Jersey cattle**											
TMSB4X	5.724	0.0002	0.004	SLC27A6	6.504	0.0003	0.005	CD69	7.777	5E-05	0.001
IL1A	5.131	5E-05	0.0014	SLC25A21	5.843	0.001	0.019	P2RY14	7.148	0.001	0.015
SPATA3	-6.557	0.004	0.036	CCDC13	5.589	0.001	0.012	ND6	6.565	0.0003	0.006
IRX1	-6.382	5E-05	0.0014	MAPK4	5.587	0.0003	0.005	KMO	6.330	0.003	0.029
SLC27A6	-6.382	0.0002	0.004	HHATL	5.512	0.002	0.021	STRA8	6.070	0.0001	0.002
SLC38A3	-6.150	0.005	0.047	SLC38A3	5.422	0.005	0.048	NFAT5	6.072	5.0E-05	0.001
SLC7A4	-6.122	5.0E-05	0.001	RUNDC3B	5.387	0.0005	0.008	TANK	5.975	5.0E-05	0.001
HPN	-5.994	5.0E-05	0.001	SCARF2	5.301	5.0E-05	0.001	CCDC146	5.928	5.0E-05	0.001
ADIRF	-5.913	0.0005	0.008	BCAS1	5.237	0.0001	0.003	CCDC83	5.772	0.0009	0.013
BDH1	-5.850	0.0002	0.004	KRT24	5.233	0.0001	0.003	BIRC2	5.744	5.0E-05	0.001

^1^Mammary epithelia cells were isolated from milk obtained from Kashmiri (n = 3) and Jersey (n = 3) cows at D15 (early lactation), D90(mid-lactation) and D250 (late-lactation) and subjected to RNA-sequencing.

^2^FDR: Benjamini and Hockberg corrected p-values

A comparison between Kashmiri vs Jersey cattle indicated that 1,103, 1,356 and 1,397 genes were differentially expressed between the two breeds on D15, D90 and D250, respectively (Fig 2c, S4 Table) while 334 DEG were common to all stages. The top 10 DEG at each lactation stage between the two breeds are shown in Table 4.

**Table 4:** Top differentially expressed genes in mammary epithelial cells with highest fold changes between Kashmiri and Jersey cattle^1^.

Stage	Genes	Log2Fold change	p-value	FDR^2^
D15	SLC18B1	9.809	0.001	0.014
	bta-mir-223	8.261	1.0E-05	0.002
	CD-207	7.411	5.0E-05	0.004
	SNORD50	-12.531	5.0E-05	0.001
	TMEM237	-9.963	5.0E-05	0.006
	TPT1	-6.971	0.003	0.037
	NOS2	6.158	0.002	0.031
	RBP4	5.906	5.0E-05	0.004
	CXCL12	-5.825	5.0E-05	0.001
	LRRC66	5.717	0.001	0.020
D90	NLRP12	8.333	0.005	0.048
	DMXL2	8.260	5.0E-05	0.001
	MGLL	7.729	5.0E-05	0.011
	STEAP4	7.394	5.0E-05	0.001
	GPR84	7.318	5.0E-05	0.001
	CXCL12	-7.173	0.005	0.048
	TMSB4X	6.952	0.0009	0.013
	MCEMP1	6.920	5.0E-05	0.001
	B3GNT6	6.798	0.001	0.019
	OCSTAMP	6.798	5.0E-05	0.001
D250	NLRP12	8.906	0.0004	0.007
	bta-mir-223	8.360	0.001	0.019
	PTX3	8.334	0.005	0.049
	GPR84	8.148	5.0E-05	0.001
	STEAP4	7.895	5.0E-05	0.001
	G0S2	7.797	5.0E-05	0.001
	BCL2A1	7.769	5.0E-05	0.001
	TIAM2	7.491	0.0005	0.008
	SNORA17	7.362	0.0002	0.004
	TG	7.346	0.001	0.021

^1^Mammary epithelia cells were isolated from milk obtained from Kashmiri (n = 3) and Jersey (n = 3) cows at D15 (15 days in milk) (early lactation), D90 (mid-lactation) and D250 (late-lactation) and subjected to RNA-sequencing.

^2^FDR: Benjamini and Hockberg corrected p-values

### Candidate genes related to milk quality and yield traits

The expression levels of about 42 genes for milk traits (fat, protein and milk yield traits) in Kashmiri and Jersey cattle are shown in Table 5. It was observed that the major candidate genes for fat synthesis like *GPAM*, *ABCG2*, *ACSS2*, *FABP3*, *THRSP*, *FASN*, *SPHK2* and *BDH1* showed higher up-regulation at D250 (D90 vs D250) in both Kashmiri and Jersey cattle. The major milk protein genes (*CSN1S1*, *CSN1S2*, *CSN2*, *CSN3*, *LALBA* and *LGB* showed up-regulated expression at D250 (D15 vs D250 and D90 vs D250) in both breeds and genes responsible for milk yield like *SLC2A4* was highly expressed at D90 (D15 vs D90) and D250 (D15 vs D250) in Kashmiri cattle and *SLC2A1* at D250 (D15 vs D250) in Jersey cattle (Table 5).

**Table 5:** Differentially expressed milk candidate genes in mammary epithelial cells for milk quality and yield traits between different stages of lactation in Kashmiri and Jersey^1^.

Genes	Kashmiri cattle (Log2Fold change)			Jersey (Log2Fold change)		
	D15 vs D90	D90 vs D250	D15 vs D250	D15 vs D90	D90 vs D250	D15 vs D250
*LPL*	-2.724	0.842	-1.881	0.028	-0.268	-0.24
*GPAM*	-3.258	1.993	-1.264	-3.694	3.73	0.035
*VLDLR*	-1.825	1.999	0.174	0.506	0.64	1.147
*DGAT1*	0.16	-1.014	-0.853	-0.184	0.473	0.289
*ABCA1*	1.477	2.015	3.492	1.168	-0.424	0.744
*LPIN1*	-3.204	1.287	-1.917	0.05	0.906	0.957
*ABCG2*	-2.178	1.251	-0.927	-3.76	3.929	0.16
*INSIG1*	-0.036	-0.301	-0.337	0.858	0.255	1.114
*ACSS1*	-1.258	-0.238	-1.496	-2.283	0.095	-2.187
*INSIG2*	0.505	0.111	0.617	1.565	-0.344	1.22
*ACSS2*	-1.859	1.834	-0.024	-2.762	2.558	-0.211
*SCAP*	0.211	-1.124	-0.913	-0.967	-0.727	-1.694
*ACSL1*	-1.035	1.346	0.311	0.521	0.333	0.854
*SREBF1*	-1.441	-0.346	-1.788	-1.085	0.529	-0.556
*SREBF2*	0.607	-0.849	-0.241	0.242	0.224	0.466
*FABP3*	-2.29	1.638	-0.652	-3.9	3.218	-0.682
*THRSP*	-1.005	1.792	0.786	-3.396	3.451	0.055
*PPARG*	4.542	0.55	5.092	1.01	-0.738	0.272
*ACACA*	-3.482	2.24	-1.241	-3.72	2.546	-1.173
*PPARGC1A*	0.543	2.242	2.785	-2.58	3.503	0.922
*FADS1*	-0.808	0.725	-0.082	1.83	-0.525	1.305
*PPARGC1B*	1.585	0.307	1.893	-0.035	0.546	0.51
*FADS2*	0.521	0.4	0.922	2.094	-1.832	0.262
*FASN*	-2.541	2.166	-0.374	-4.247	4.523	0.275
*SPTLC1*	-0.243	0.862	0.619	1.159	0.753	1.912
*SPTLC2*	0.739	0.06	0.8	0.688	0.024	0.712
*SPHK2*	-1.347	0.817	-0.529	-1.157	0.224	-0.933
*XDH*	-2.595	2.311	-0.284	-1.451	2.262	0.811
*SGPL1*	1.392	0.512	1.905	1.32	-0.271	1.048
*UGCG*	0.862	0.809	1.671	1.293	2.355	3.649
*OSBP*	-0.142	-0.785	-0.927	-0.523	0.022	-0.501
*BDH1*	-3.248	1.839	-1.408	-5.85	3.008	-2.842
*OSBPL2*	-1.212	0.174	-1.038	-0.11	0.401	0.29
*OXCT1*	0.161	0.757	0.919	0.756	0.223	0.98
*CSN1S1*	-0.238	0.598	0.36	-0.07	0.581	0.511
*CSN1S2*	0.33	0.485	0.815	0.15	0.124	0.275
*CSN3*	-0.021	0.27	0.248	-0.268	0.617	0.349
*CSN2*	-0.189	0.295	0.106	-0.283	0.293	0.009
*LGB*	-0.588	0.5956	0.007	0.957	0.698	1.656
*LALBA*	0.148	0.48	0.629	-0.037	0.173	0.135
*HK2*	0.355	1.232	1.587	-1.157	0.224	-0.933
*SLC2A1*	-1.006	0.456	-0.549	0.995	0.44	1.435
*SLC2A4*	1.655	0.48	2.136	-1.311	-0.377	-1.688
*SLC2A8*	-0.986	0.101	-0.884	-1.506	-0.902	-2.408

^1^Mammary epithelia cells were isolated from milk obtained from Kashmiri (n = 3) and Jersey (n = 3) cows at D15 (early lactation), D90 (mid-lactation) and D250 (late-lactation) and subjected to RNA-sequencing.

### Protein-protein interaction

**Fig 3:**
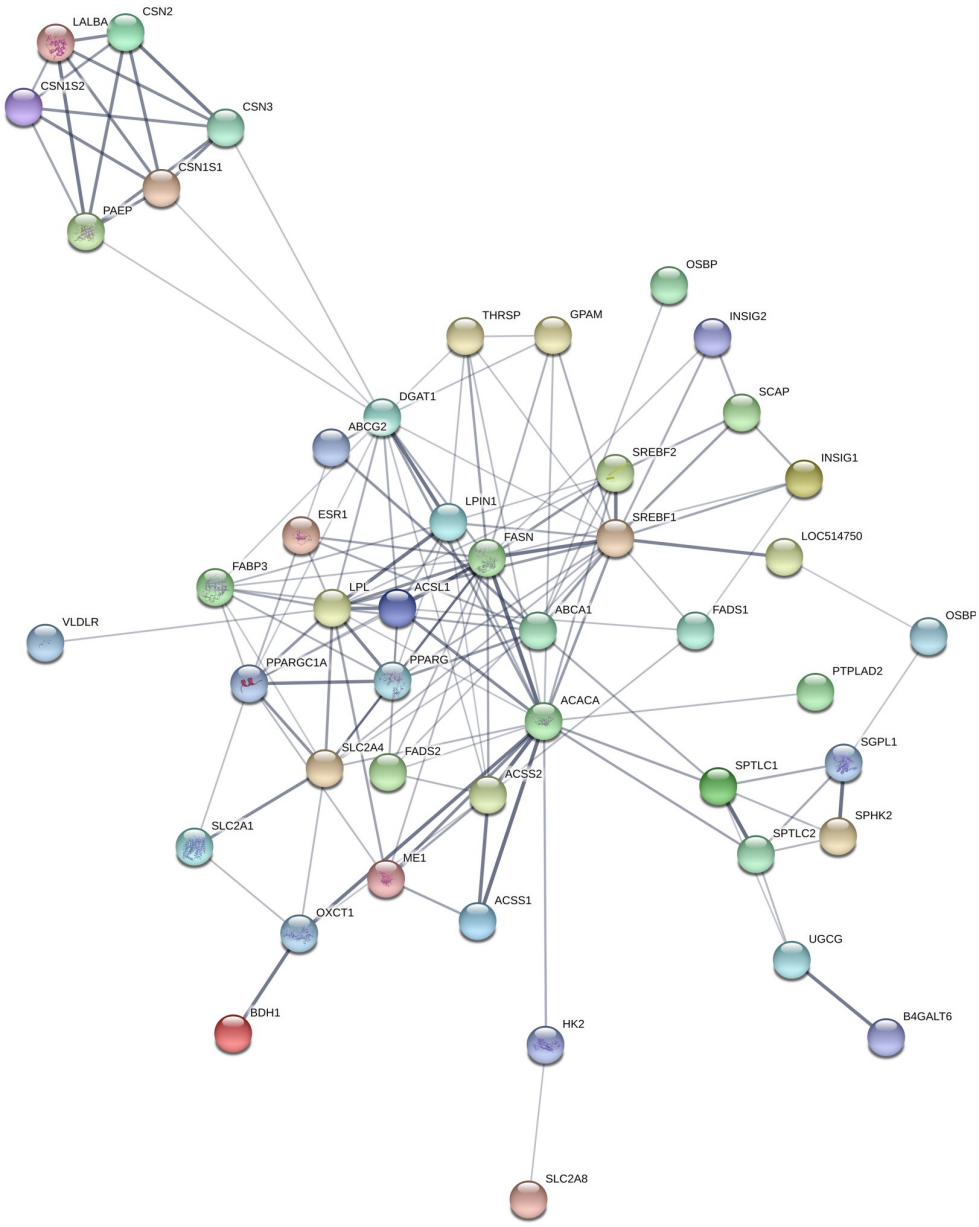
Protein-protein interactions among top 20 differentially expressed candidate genes and differentially expressed genes for milk traits from lactation stage comparisons in Kashmiri cattle. In the network view, nodes are proteins while edges represent predicted functional interactions; the low interaction nodes are hidden.

**Fig 4:**
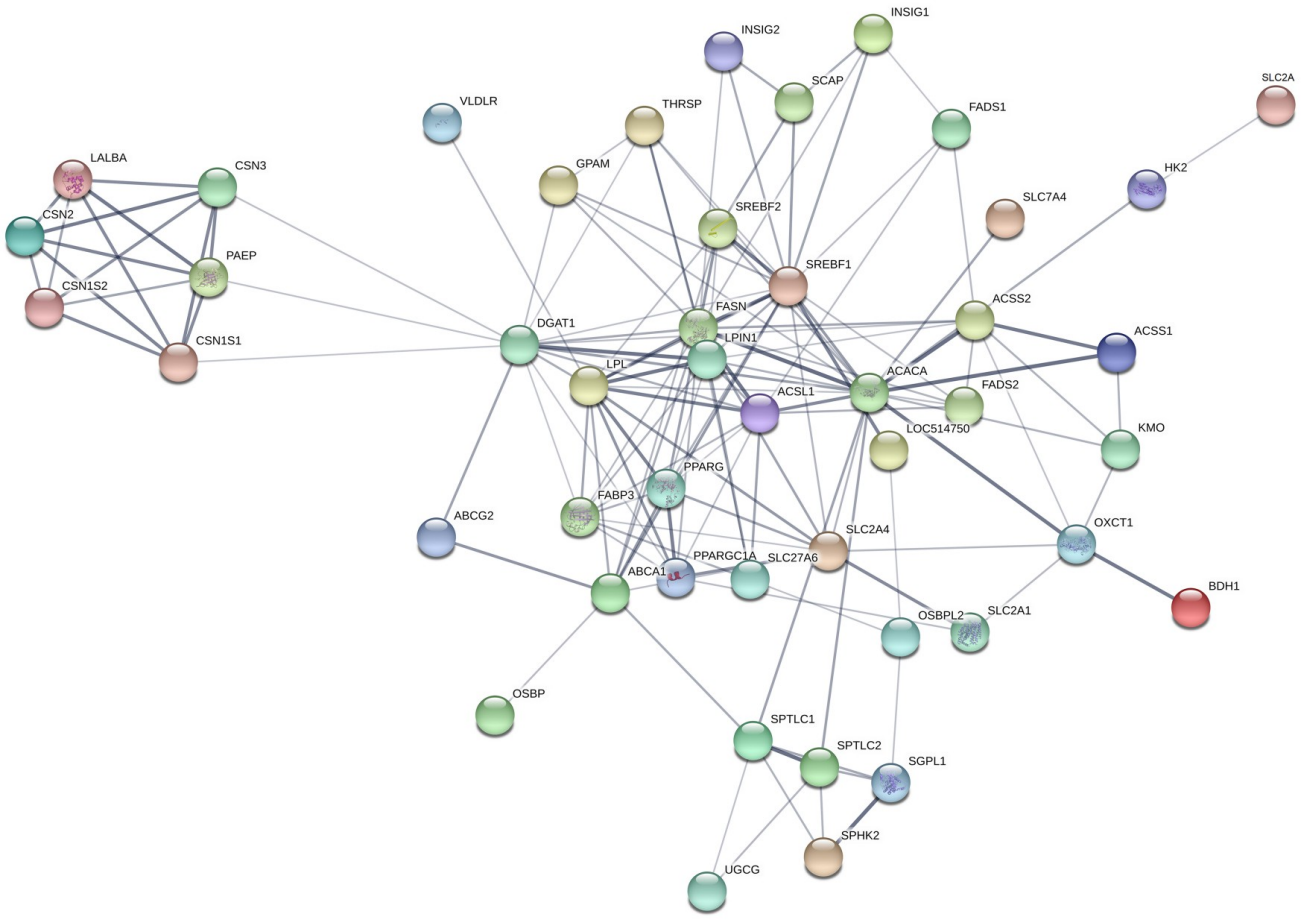
Protein-protein interactions among top 20 differentially expressed candidate genes and differentially expressed genes for milk traits from lactation stage comparisons in Jersey cattle. In the network view, nodes are proteins while edges represent predicted functional interactions; the low interaction nodes are hidden.

We examined possible interactions between top 20 differentially expressed genes (Table 3 and S4 Table) and candidate genes for milk yield and quality traits (Table 5) at each lactation stage comparisons (D15 vs D90, D90 vs D250 and D15 vs D250) in Kashmir and Jersey cattle using the STRING database [26]. Out of 60 DEG (20 top genes from each lactation stage comparison) in Kashmiri cattle, only *ME1*, *B4GALT6* and *ESR1* showed protein interactions with major milk candidate genes (Fig 3) with interaction confidence > 0.4. Whereas in Jersey cattle, *SLC27A6*, *BDH1* and *KMO* showed multiple interactions with various milk candidate genes (Fig 4). The details of string analysis are shown in supplementary file (S5 Table).

### Gene ontology and pathway enrichment analysis of differentially expressed genes

According to lactation stage comparisons, a total of 12 (localization, biological regulation, response to stimulus, multicellular organismal process, endopeptidase activity, binding, receptor activity, signal transducer activity, catalytic activity, transporter activity, cell junction and macromolecular complex), 5 (cellular process, developmental process, biological adhesion, cell junction and macromolecular complex) and 4 (biological regulation, response to stimulus, multicellular organismal process and extracellular region) GO terms were significantly enriched (FDR < 0.05) by DEG at D15 vs D90, D90 vs D250 and D15 vs D250, respectively in Kashmiri cattle (Table 6 and S6 Table). Similarly, 7 (reproduction, developmental process, multicellular organismal process, locomotion, receptor activity, signal transducer activity and catalytic activity), 3 (signal transducer activity, synapse and macromolecular complex) and 10 (localization, reproduction, multicellular organismal process, metabolic process, immune system process, receptor activity, signal transducer activity, catalytic activity, cell part and organelle) GO terms were significantly enriched (FDR < 0.05) by DEG at D15 vs D90, D90 vs D250 and D15 vs D250, respectively in Jersey cattle (Table 6 and S6 Table). Only 6 GO terms (multicellular organismal process, receptor activity, signal transducer activity, catalytic activity, macromolecular complex and multicellular organismal process) were commonly enriched by DEG in Kashmiri and Jersey cattle.

**Table 6:** Significantly enriched gene ontology (GO) terms associated with identified differentially expressed genes in mammary epithelial cells in Kashmiri and Jersey cattle^1^.

Comparison	Kashmiri						Jersey					
	GO term	GO ID	Genes	FE	P-value	FDR^2^	GO term	GO ID	Genes	FE	P-value	FDR
**D15 VS D90**	**Biological function**											
	Localization	GO:0051179	188	1.26	0.002	**0.019**	Reproduction	GO:0000003	3	0.22	0.001	**0.017**
	Biological regulation	GO:0065007	233	0.69	4.0E-07	**2.0E-05**	Developmental process	GO:0032502	102	1.33	0.005	**0.047**
	Response to stimulus	GO:0050896	157	0.69	4.0E-07	**2.0E-05**	Multicellular organismal process	GO:0032501	52	0.6	7.4E-05	**0.003**
	Multicellular organismal process	GO:0032501	91	0.67	5.2E-05	**0.001**	Locomotion	GO:0040011	27	2.09	6.4E-04	**0.013**
	Endopeptidase activity	GO:0010950	3	1.24	0.000	**0.050**	Metabolic process	GO:0008152	307	1.14	0.012	0.141
	**Molecular function**											
	Binding	GO:0005488	303	0.83	3.2E-04	**0.006**	Receptor activity	GO:0004872	39	0.54	1.7E-05	**0.001**
	Receptor activity	GO:0004872	62	0.54	1.0E-07	**9.6E-06**	Signal transducer activity	GO:0004871	37	0.6	8.7E-04	**0.021**
	Signal transducer activity	GO:0004871	51	0.52	3.7E-07	**2.4E-05**	Catalytic activity	GO:0003824	258	1.21	9.3E-04	**0.020**
	Catalytic activity	GO:0003824	408	1.22	2.8E-05	**8.9E-04**	Antioxidant activity	GO:0016209	5	3.23	0.026	0.261
	Transporter activity	GO:0005215	100	1.36	0.004	**0.050**	-	-	-	-		-
	**Cellular component**											
	Synapse	GO:0045202	9	2.05	0.051	0.134	Synapse	GO:0045202	8	2.88	0.010	0.318
	Cell junction	GO:0030054	17	2.3	0.003	**0.020**	Extracellular region	GO:0005576	41	1.43	0.034	0.267
	Macromolecular complex	GO:0032991	122	0.71	6.9E-05	**0.001**	-	-	-	-		-
**D90 VS D250**	**Biological function**											
	Cellular process	GO:0009987	278	1.22	2.6E-05	**6.2E-04**	Localization	GO:0051179	71	1.43	0.003	0.134
	Developmental process	GO:0032502	86	1.98	2.9E-09	**7.2E-07**	Multicellular organismal process	GO:0032501	29	0.64	0.011	0.228
	Rhythmic process	GO:0048511	2	7.05	0.041	0.227						
	Biological adhesion	GO:0022610	27	3.25	2.8E-07	**1.4E-05**	-	-	-	-	-	-
	Locomotion	GO:0040011	15	2.06	0.013	0.103	-	-	-	-	-	-
	**Molecular function**											
	Binding	GO:0005488	153	1.17	0.034	0.269	Receptor activity	GO:0004872	22	0.58	0.005	0.129
	Receptor activity	GO:0004872	28	0.68	0.032	0.266	Signal transducer activity	GO:0004871	13	0.4	1.4E-04	**0.026**
	Structural molecule activity	GO:0005198	28	1.81	0.004	0.06	Transporter activity	GO:0005215	39	1.59	0.007	0.154
	Signal transducer activity	GO:0004871	22	0.63	0.021	0.22	-	-	-	-	-	-
	**Cellular component**											
	Synapse	GO:0045202	5	3.18	0.025	0.158	Synapse	GO:0045202	6	4.1	0.005	**0.050**
	Cell junction	GO:0030054	21	7.91	4.8E-12	**2.9E-10**	Macromolecular complex	GO:0032991	31	0.54	1.1E-04	**0.007**
	Macromolecular complex	GO:0032991	39	0.63	0.002	**0.024**	Extracellular matrix	GO:0031012	7	2.65	0.020	0.106
**D15 VS D250**	**Biological function**											
	Biological regulation	GO:0065007	118	0.74	3.0E-04	**0.009**	Cellular process	GO:0009987	951	1.07	0.013	0.066
	Response to stimulus	GO:0050896	75	0.63	5.0E-06	**2.0E-04**	Localization	GO:0051179	271	1.3	3.9E-05	**7.4E-04**
	Multicellular organismal process	GO:0032501	44	0.61	4.7E-04	**0.013**	Reproduction	GO:0000003	15	0.49	0.003	**0.023**
	Biological adhesion	GO:0022610	21	1.72	0.0197	0.178	Response to stimulus	GO:0050896	283	0.89	0.038	0.148
	positive regulation of peptidase activity	GO:0010952	3	1.20	0.000	0.081	Multicellular organismal process	GO:0032501	91	048	2.0E-15	**1.2E-13**
	-	-	-	-	-	-	Metabolic process	GO:0008152	718	1.2	7.7E-08	**2.3E-06**
	-	-	-	-	-	-	Immune system process	GO:0002376	49	1.56	0.006	**0.035**
	**Molecular function**											
	Binding	GO:0005488	160	0.83	0.008	0.141	Binding	GO:0005488	560	1.09	0.022	0.147
	Receptor activity	GO:0004872	36	0.6	7.6E-04	**0.029**	Receptor activity	GO:0004872	98	0.61	1.8E-07	**1.7E-05**
	Structural molecule activity	GO:0005198	33	1.45	0.041	0.365	Signal transducer activity	GO:0004871	70	0.51	6.5E-10	**1.2E-07**
	Transporter activity	GO:0005215	52	1.34	0.045	0.388	Catalytic activity	GO:0003824	570	1.21	1.7E-06	**1.0E-04**
	**Cellular function**											
	Extracellular matrix	GO:0031012	9	2.16	0.042	0.242	Macromolecular complex	GO:0032991	273	1.14	0.035	0.131
	Extracellular region	GO:0005576	42	1.76	7.6E-04	**0.012**	Cell part	GO:0044464	732	1.11	0.001	**0.013**
	-	-	-	-	-	-	Organelle	GO:0043226	464	1.14	0.004	**0.051**

^1^Mammary epithelia cells were isolated from milk obtained from Kashmiri (n = 3) and Jersey (n = 3) cows at D15 (15 days in milk) (early lactation), D90 (mid-lactation) and D250 (late-lactation) and subjected to RNA-sequencing.

^2^FDR: Benjamini and Hockberg corrected p-values

Pathway enrichment analysis at different stages of lactation indicated that 5 pathways (transcription regulation by bZIP transcription factor, Toll receptor signalling pathway, VEGF signalling pathway, CCKR signalling pathway and chemokine and cytokine signalling pathway) and 11 pathways (JAK/STAT signalling pathway, p38 MAPK pathway, B cell activation, Toll receptor signalling pathway, interleukin signalling pathway, apoptosis signalling pathway, inflammation mediated by chemokine and cytokine signalling, phosphatidylinositol-3-kinases (PI3) pathway, Platelet-derived growth factor (PDGF) signalling pathway, T cell activation and cholecystokinin receptor (CCKR) signalling pathway were significantly enriched (FDR < 0.05) by DEG of D15 vs D90 and D15 vs D250, respectively in Jersey cattle while only two pathways, purine metabolism (FDR = 0.095) and p38 MAPK pathway (FDR = 0.063) (D15 vs D90) tended towards significance in Kashmiri cattle (Table 7 and S7 Table).

**Table 7:** Enriched KEGG pathways for differentially expressed genes in mammary epithelial cells between lactation stages in Kashmiri and Jersey cattle^1^.

Kashmiri						Jersey					
Pathway	ID	Genes	Fold enrichment	P-value	FDR^2^	Pathway	ID	Genes	Fold enrichment	P-value	FDR
**D15 VS D90**											
Purine metabolism	P02769	6	7.6	0.000	0.095	Salvage pyrimidine deoxyribonucleotides	P02774	2	14.63	0.017	0.258
2-arachidonoylglycerol biosynthesis	P05726	3	5.97	0.025	0.685	Cholesterol biosynthesis	P00014	4	5.85	0.008	0.149
Pyrimidine Metabolism	P02771	5	4.35	0.011	0.451	Plasminogen activating cascade	P00050	5	4.99	0.005	0.132
Cholesterol biosynthesis	P00014	4	3.71	0.034	0.706	Axon guidance mediated by Slit/Robo	P00008	4	3.99	0.025	0.313
p38 MAPK pathway	P05918	11	3.56	0.000	0.063	Transcription regulation by bZIP transcription factor	P00055	11	3.66	0.000	**0.0199**
Axon guidance mediated by Slit/Robo	P00008	5	3.16	0.031	0.733	p38 MAPK pathway	P05918	7	3.57	0.005	0.116
Blood coagulation	P00011	9	2.67	0.011	0.378	Toll receptor signalling pathway	P00054	10	3.48	0.001	**0.033**
Hypoxia response via HIF activation	P00030	6	2.46	0.048	0.793	VEGF signalling pathway	P00056	11	3.26	0.001	**0.037**
Apoptosis signalling pathway	P00006	3	0.31	0.037	0.670	Enkephalin release	P05913	5	3.05	0.031	0.346
-	-	-	-	-		CCKR signalling map	P06959	23	2.64	0.000	**0.003**
-	-	-	-	-		Inflammation mediated by chemokine and cytokine signalling pathway	P00031	29	2.58	0.000	**0.002**
-	-	-	-	-		Apoptosis signalling pathway	P00006	13	2.13	0.013	0.222
-	-	-	-	-		p53 pathway	P00059	9	2.12	0.044	0.427
-	-	-	-	-		EGF receptor signalling pathway	P00018	13	1.88	0.034	0.347
-	-	-	-	-		Angiogenesis	P00005	15	1.87	0.029	0.346
**D90 VS D250**											
Purine metabolism	P02769	2	7.05	0.041	0.668	Pyrimidine Metabolism	P02771	3	7.82	0.009	0.305
p38 MAPK pathway	P05918	4	3.61	0.030	0.613	Purine metabolism	P02769	2	7.59	0.036	0.651
T cell activation	P00053	9	3.23	0.002	0.229	Plasminogen activating cascade	P00050	4	7.59	0.002	0.240
EGF receptor signalling pathway	P00018	11	2.81	0.00284	0.154	Angiotensin II-stimulated signalling through G proteins and beta-arrestin	P05911	5	5.09	0.004	0.233
Cadherin signalling pathway	P00012	10	2.73	0.005	0.169	Interferon-gamma signalling pathway	P00035	3	4.64	0.032	0.661
B cell activation	P00010	5	2.69	0.044	0.660	Inflammation mediated by chemokine and cytokine signalling pathway	P00031	16	2.7	0.000	0.0827
p53 pathway	P00059	6	2.5	0.038	0.703	Cytoskeletal regulation by Rho GTPase	P00016	6	2.78	0.025	0.589
Integrin signalling pathway	P00034	11	2.41	0.008	0.223	-	-	-	-	-	
Inflammation mediated by chemokine and cytokine signalling pathway	P00031	13	2.04	0.016	0.376	-	-	-	-	-	
**D15 VS D250**											
Purine metabolism	P02769	3	7.19	0.013	0.534	JAK/STAT signalling pathway	P00038	10	4.96	0.000	**0.003**
Blood coagulation	P00011	7	3.93	0.003	0.549	ATP synthesis	P02721	4	3.97	0.033	0.226
-	-	-	-	-		p38 MAPK pathway	P05918	17	3.92	0.000	**0.000**
-	-	-	-	-		Vitamin D metabolism and pathway	P04396	6	3.72	0.012	0.116
-	-	-	-	-		B cell activation	P00010	25	3.45	0.000	**0.000**
-	-	-	-	-		Cholesterol biosynthesis	P00014	5	3.31	0.030	0.219
-	-	-	-	-		Toll receptor signalling pathway	P00054	20	3.15	0.000	**0.000**
-	-	-	-	-		Interferon-gamma signalling pathway	P00035	8	2.94	0.012	0.110
-	-	-	-	-		Interleukin signalling pathway	P00036	27	2.88	0.000	**0.000**
-	-	-	-	-		Apoptosis signalling pathway	P00006	36	2.67	0.000	**0.000**
-	-	-	-	-		Inflammation mediated by chemokine and cytokine signalling pathway	P00031	64	2.57	0.000	**0.000**
-	-	-	-	-		PI3 kinase pathway	P00048	16	2.56	0.002	**0.0257**
-	-	-	-	-		PDGF signalling pathway	P00047	38	2.48	0.000	**0.000**
-	-	-	-	-		T cell activation	P00053	26	2.39	0.000	**0.003**
-	-	-	-	-		CCKR signalling map	P06959	43	2.23	0.000	**0.000**
-	-	-	-	-		Ubiquitin proteasome pathway	P00060	14	2.2	0.014	0.128
-	-	-	-	-		Ras Pathway	P04393	16	2.12	0.009	0.106
-	-	-	-	-		Transcription regulation by bZIP transcription factor	P00055	14	2.1	0.017	0.142
-	-	-	-	-		Integrin signalling pathway	P00034	29	1.63	0.020	0.150

^1^Mammary epithelia cells were isolated from milk obtained from Kashmiri (n = 3) and Jersey (n = 3) cows at D15 (15 days in milk) (early lactation), D90 (mid-lactation) and D250 (late-lactation) and subjected to RNA-sequencing.

^2^FDR: Benjamini and Hockberg corrected p-values

### Real time quantitative PCR validation of the RNA-seq expression results

**Fig 5:**
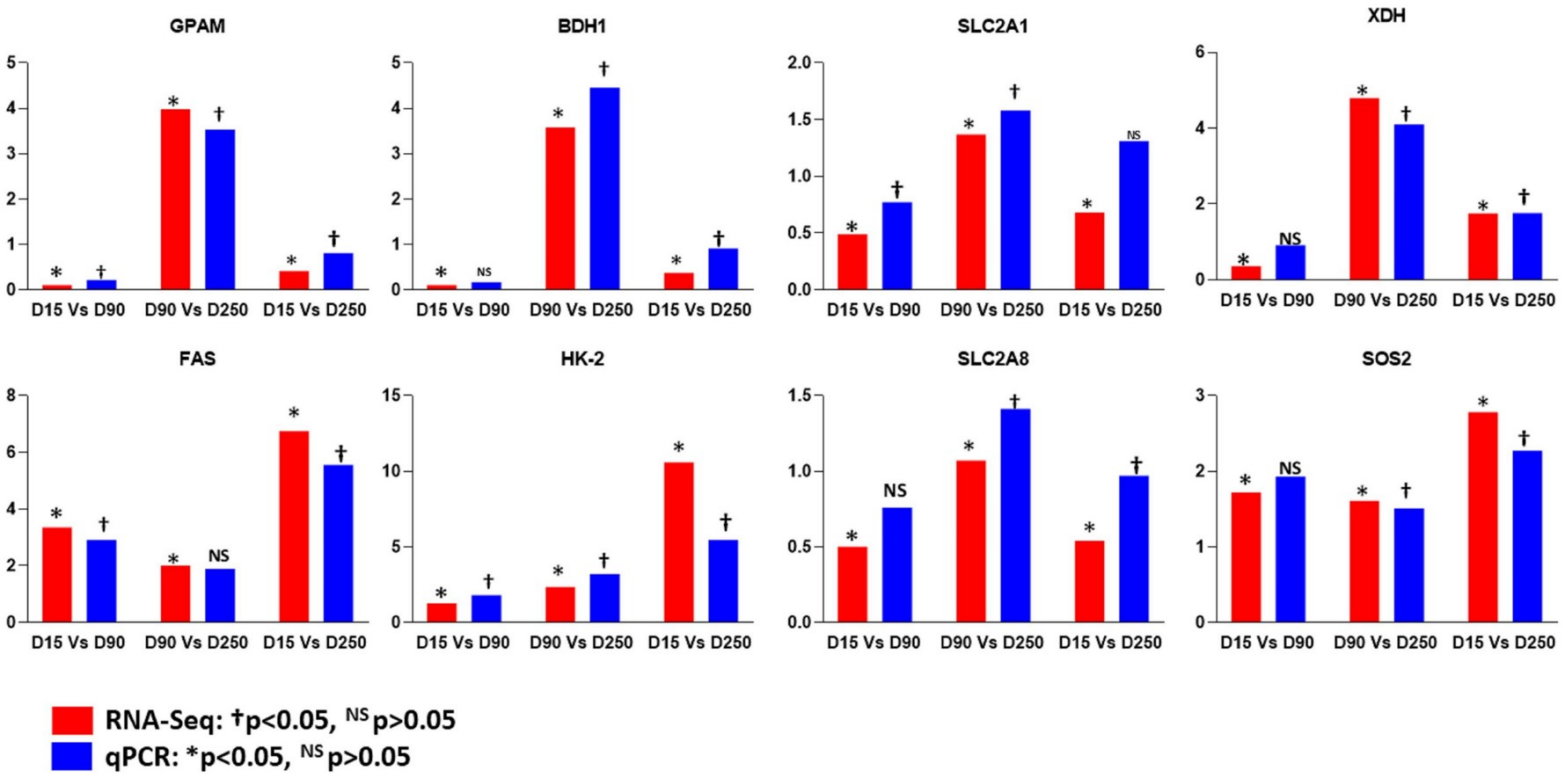
Comparison of the expression levels of eight differentially expressed genes obtained by RNA-seq and qPCR detection methods.

Results of qPCR analysis of the expression of 8 DEG involved in different milk synthesis pathways (*GPAM*, *BDH1*, *SLC2A1*, *SLC2A8*, *HK-2*, *FAS*, *SOS2* and *XDH)* and data obtained by RNA-Seq are shown in Fig 5. The expression levels of genes by qPCR and RNA-Seq were highly correlated (Pearson’s correlation coefficient = 0.87) thus validating the RNA-Seq results.

## Discussion

In this study, we investigated the transcriptome profile of bovine MEC at different stages of lactation in Kashmiri and Jersey cattle by the method of high throughput RNA sequencing. Purified RNA from MEC, which represents a non-invasive source of material for assessing gene expression in mammary gland [24] was used. The quality of RNA from milk isolated MEC can be sensitive to degradation (due to several long processing steps) resulting in a wide range of RIN values (4 to 9) [24,31,32]. Using RNA from MEC with RIN of 6, Canovas et al. [24] reported discrepancies in gene expression when compared with other sources of mammary RNA (mammary gland tissue, milk somatic cells, laser micro dissected mammary epithelial cells and milk fat globules) [24]. Consequently, Boutinaud *et al*. [17] advised that the quality of isolated MEC RNA should be assessed before use in gene expression analysis. In this study, the RIN values of isolated MEC RNA ranged from 7.4 to 9.1 suggesting that the RNA was of high quality with minimal degradation. Therefore, the low RIN 6 reported by Canovas *et al*. [24] could explain the relatively low levels of *CSN2*, *CSN3*, *CSN1S1* and *CSN2S2* when compared with our data, suggesting that the antibody-captured milk MEC technique used by these authors was probably not optimal. The validity of gene expression results obtained by using purified MEC has been demonstrated in cows and buffalo [31, 33–36] and supported by our data. The advantages of purified MEC as a non-invasive source of RNA for mammary gland transcriptome analysis include but not limited to possibility for repeat sampling over a period of time on the same animals without causing damage to mammary tissue and ability to specifically study milk secreting cells.

The qPCR results of *CSN2*, *KRT18*, *LSP1*, *HBA* and *CD18* suggest that the purified MEC in this study share characteristics with typical mammary gland epithelial cells and were minimally contaminated with other cell types like macrophages, leucocytes and red blood cells as we detected very low mRNA abundance of *LSP1*, *HBA* and *CD18* in isolated MEC (Fig 1). However, our results contrast findings by Cánovas et al. [24] who showed the possibility of higher contamination of purified MEC by macrophages, further supporting our suggestion that the antibody-captured milk MEC technique was not optimally applied in that study.

The RNA sequencing results were validated by real time qPCR of eight genes which showed high correlation (r = 0.87) in their expression levels with RNA sequencing data. Further validation of DEG in milk synthesis related pathways between the two breeds in a larger population is necessary. Such validation could not be performed within this study due to the limited sample size. The transcriptome results revealed that the majority of genes were lowly expressed (FPKM<10) (S3 Table). This observation is in agreement with previous reports on the mammary gland transcriptome of Canadian Holsteins and transgenic goats [10,37,38]. The read counts of highly expressed genes (Table 2) constituted about 70% of total read counts in both breeds. The caseins (*CSN1S1*, *CSN1S2*, *CSN3*, *CSN2*) and whey protein genes (*LGB*, *LALB*) were amongst the top expressed genes as expected. Our observation is in agreement with Ibeagha-Awemu et al. [38].

The DEG profile between lactation stages in this study followed the dynamics of a typical lactation curve. It was found that the highest numbers of genes were expressed in early lactation in both breeds. A higher protein and fat content observed in this study during the initial stages of lactation and supported by a previous report [39] could be a possible reason for this observation. Casein and whey protein gene expression remained almost constant throughout lactation in both breeds. However, the expression was higher in Jersey cattle as compared to Kashmiri cattle. It is likely that the casein and whey protein genes have been fixed through long term genetic selection for increased milk production and consequently accounted for their increased frequencies in Jersey cattle. Apart from the casein and whey protein genes, other genes like *RPLP1*, *RPS28*, *RPLPO* and *B2M* were also highly expressed in the two breeds. *RPLP1* gene encodes a ribosomal phosphoprotein that is a component of the 60S ribosomal subunit and plays an important role in the synthesis of proteins, protein folding and transport [40]. Deficiency of RPS28 and RPLPO proteins has been shown to cause cell death through reactive oxygen species (ROS) accumulation and MAPK1/ERK2 signalling pathway activation [41]. In this study, *RPS28* was highly expressed during all three lactation stages in Kashmiri cattle while in Jersey, it was expressed only at early lactation. Similarly, *RPLPO* was highly expressed during early and late lactation in Kashmiri cattle and during early lactation in Jersey cows. The expression patterns of *RPS28* and *RPLPO* suggests a possible higher antioxidant activity of milk from Kashmiri cattle as compared to Jersey cattle. *B2M* gene is among highly expressed genes (higher expression in Jersey cattle) and encodes for the beta-2-microglobulin protein, an integral component of the Fc receptor heterodimer involved in transferring IgG from blood into milk across mammary epithelial cells [42]. Some *B2M* haplotypes have been reported to be related to higher concentrations of IgG1 in bovine milk [43]. Increasing IgG levels in milk could become important as IgG enhanced dairy products are in demand by consumers to obtain protective immunity [44]. *CCL14*, a small cytokine belonging to the CC chemokine family was highly expressed during mid and late lactation stages in Jersey cows. *CCL14* is involved in cellular calcium homeostasis, immune response and positive regulation of cell proliferation [45].

Differential gene expression analysis indicated that more genes were differentially expressed in Jersey as compared to Kashmiri cattle. In Kashmiri cattle, only three genes, *SNORD50*, *PEAR1* and *TMEM232*, were amongst top DEG between lactation stages. The membrane protein, *PEAR1*, shows specific expression in endothelial and platelets cells and plays significant roles in platelet activity and cardiovascular disease [46,47]. *TMEM232* has been reported to be co-expressed with *SLC25A46* and *NAA1*0. Through genome wide association studies, *TMEM232* along with *SLC25A46* have been found to dysregulate IgE concentration [48] and are associated with various allergic conditions, while *NAA10* was found to regulate mTOR pathway which regulates milk protein synthesis [49].

In Jersey, *TMSB4X*, which was highly up-regulated at D90, is involved in T-cell activation [50], pathogen clearance and anti-inflammatory effects [51]. *SLC27A6* was found to be highly up-regulated during early and late lactation in Jersey and is involved in fatty acid uptake by mammary epithelial cells. *SLC27A6* is the major isoform of solute carrier group of genes expressed in bovine MEC and its expression was highly up-regulated with the onset of lactation [52,53].

The expression of all the top DEG between Kashmiri and Jersey cattle (Table 4) were higher in Jersey except *SNORD50* and *TMEM237*, which were expressed at a higher rate in Kashmiri cattle during early lactation. *Bos taurus* microRNA-223 (bta-miR-223) was differentially expressed between Kashmiri and Jersey and has known roles in immunity, immune cell lineage differentiation, and granulopoiesis [53]. Bta-miR-223 has been implicated in cancer progression, HIV-1 infection, IL-17–induced inflammation [54,55], general delay in alternative NF-κB activation in innate immune cells [56] and negative regulation of proliferation and differentiation of neutrophils through down-regulation of the transcription factor, Mef2c, as well as up-regulation during bovine mammary gland infections like mastitis [57]. *SNORD50* is involved in maturation of ribosomal RNAs (rRNAs) within the nucleolus [58,59] and its overexpression inhibits colony formation of human breast and prostate cancer cell lines *in vitro*, suggesting its role as a tumor suppressor [60,61]. *PTX3* play roles in the regulation of resistance to pathogens, inflammatory reaction, clearance of self-components and female fertility [62,63,64]. *GPR84* is expressed mainly in immune-related tissues and plays significant roles in inflammatory processes [65]. It is also expressed in adipose tissue [66]. It enhances insulin secretion from pancreatic β-cells [67,68] and increases the release of gut peptides, glucagon-like peptide 1 from intestinal neuroendocrine L-cells [69].

The candidate genes for milk quality and yield traits a were expressed at higher rates in Jersey cattle as compared to Kashmiri cattle (Tables 4 and 5). This observation is supported by milk yield, and milk fat and protein contents which were higher in Jersey at all stages of lactation as compared to Kashmiri cattle. Similar differential expression patterns were also reported by Lee et al., [70] in lactating yaks.

The relative expression of the major milk protein genes (*CSN1S1*, *CSN1S2*, *LALBA CSN3*, *CSN2* and *LGB*) showed highest fold changes at D250 (late-lactation) in both Kashmiri and Jersey cattle. Our findings are in agreement with Sigl et al. [33] and Colitti and Farinacci [71]. Similar to our results, Colitti and Pulina [72] recorded higher expression for *CSN1S1*, *CSN3* and *CSN2* during late lactation in dairy ewes. Amongst the glucose transporter genes (*SLC2A1*, *SLC2A4* and *SLC2A8*, and *HK2*), only *SLC2A1* was up-regulated at mid-lactation in Kashmiri cattle which could be attributed to increased demand for glucose during mid-lactation period [73]. *SLC2A8*, another major solute carrier that plays a significant role in glucose transport in mammary gland indicated higher expression during mid-lactation (D90). *SLC2A8* mRNA in mammary gland is developmentally regulated during lactation in both mouse and cows [74]. *SLC2A4* expression, unlike *SLC2A1* expression was found to be higher during late lactation and could influence the insulin action on mammary tissue during involution [75].

Data on protein-protein interaction indicated that ME1 protein had varying degrees of interactions with milk candidate genes (*ACSS1*, *ACSS2*, *PPARGC1A*, *LPL* and *FSN*) with critical roles in metabolic pathways, insulin signalling, fatty acid synthesis and metabolism. *ME1* gene encodes a cytosolic NADP-dependent enzyme that generates NADPH used by *FASN* for long chain fatty acid synthesis [76]. *B4GALT6* interacted (confidence score = 0.905) with *UGCG* milk candidate gene. *B4GALT6*, a galactosyltransferase gene of the sphingolipid metabolic pathway encodes for a lactosyl ceramide synthase enzyme that is required for cell apoptosis [77] and lactose biosynthesis (occurs exclusively in the mammary gland) [78]. ESR1 protein interacted with lipogenic proteins (LPL, ABCA1, FASN and PPARGC1A) with confidence scores varying from 0.5 to 0.61. ESR1 plays a key regulatory role in lipid biosynthesis in mammary epithelial cells through activation of various lipogenic genes (*SREBF1*, *SREBF2*, *PPARG*, *INSIG1*, and *PPARGC1A*) [79]. *SLC27A6* interacted with *ACSL1* and *LPIN1* with confidence score of 0.63 and 0.66, respectively. SLC27A6 is an integral transmembrane protein that enhance the uptake of long-chain and very long chain fatty acids into cells by activating LC-acyl-CoA primarily via *ACSL1* [80]. BDH1 showed protein-protein interaction only with OXCT1 (confidence score > 0.9). Both BDH1 and *OXCT1* are involved in ketone body utilization through synthesis and degradation of ketone bodies pathway, which utilize β-hydroxybutyrate for *de novo* fatty acid synthesis in mammary epithelial cells [81].

In mammals, the maintenance of lactogenic process is because of the balance between different processes like mammary development and involution pathways [82]. In relation to these physiological processes, many of the enriched GO terms in our data are related to developmental processes (GO:0032502). With respect to involution, this mechanism produces changes in mammary gland architecture through extra cellular matrix remodelling, collapse of alveoli and differentiation of adipocytes [83]. Cell junction (GO:0030054) and synapse (GO:0045202) which are related to cellular components of ECM were enriched in Kashmiri and Jersey cattle, respectively. The enriched genes for these terms (*MEGF9*, *HAPLN3*, *ELN*, *LAMB3*, *GAS6*, *VCAN*, *TIMP1*) have been associated with the onset of mammary gland involution and mammary gland morphogenesis [84,85]. Our results did not show a direct relationship with prolactin signalling, which is important for the process of lactogenesis. However, a significant GO term, response to stimulus (GO:0050896), is a parent term to insulin and growth factor stimulus. It is remarkable that both insulin and growth hormone are known to increase prolactin lactogenic effect [85]. Additionally, it is important to highlight that the significant GO term related to organelle (GO:0043226) (includes endoplasmic reticulum lumen) was significantly enriched at D250 (D15 vs D250) of lactation in Jersey. This organelle is linked to the lipid secretor mechanism of mammary epithelial cells [86]. The GO term, endopeptidase activity (GO: 0010950) was highly enriched in Kashmiri cattle (D15 vs D90) and may have special effects on the physico-chemical characteristics of milk [87] and flavour of dairy products [88] in this breed. Moreover, the immune system process (GO:0002376) GO term was significantly enriched by DEG in Jersey (D15 vs D250). Subclinical infections elicit elevated somatic cell counts (SCC) but other variables like breed have been shown to influence milk SCC levels [89].

The pathway enrichment analysis indicated that a total of 16 pathways were enriched (FDR<0.05) for DEG in Jersey and only two pathways (purine metabolism and p38 MAPK pathway) tended (FDR<0.1) to be significant in Kashmiri cattle. Among enriched pathways, the bZIP transcription factor regulates the transcriptional activity of various protein coding genes like *GTF2B*, *CREB1*, *POLR2L* which play critical roles in the regulation of mammary epithelial cell proliferation [90]. P13 kinase regulates mammary epithelial cell differentiation through prolactin action. The mammary differentiation due to P13K-AKT activation results in autocrine prolactin secretion which in turn activates JAK-STAT pathway [91]. Lemay et al. [92] observed that P13K-AKT pathway was highly significantly enriched during lactation in mouse mammary gland. JAK-STAT pathway plays important roles in the regulation of milk protein synthesis in non-ruminants [93]. Besides protein synthesis, STAT3, JAK2 and JAK3 are important for mammary gland development [94]. In bovine, STAT3 responds to lactogenic factors and its activity increases during lactation [95]. The p38-MAPK pathway is another signalling pathway which plays a critical role in mammary epithelial cell development and enhances milk production by modulating alveolar cell proliferation and branching [96]. A number of immune related pathways (B cell activation, Toll receptor signalling pathway, T cell activation) were significantly enriched in Jersey cattle and may play an important role in milk production by protecting offspring and cell secreting organs [97].

## Conclusion

This study represents a cohesive comparison of the milk epithelial cell transcriptome profiles at different stages of lactation between Kashmiri and Jersey cattle. The results revealed higher gene expression profiles of candidate genes for milk synthesis and yield traits in Jersey compared to Kashmiri cattle. More genes were differentially expressed between lactation days in Jersey cattle as compared to Kashmiri cattle. Sixteen pathways were significantly enriched by DEG in Jersey cattle while no pathway was found to be significantly enriched in Kashmiri cattle. On the other hand, varied numbers of GO terms were enriched between lactation stages in both Kashmiri and Jersey cattle. The presence of enriched GO terms like endopeptidase and antioxidant activity in Kashmiri cattle suggests special effects on the physico-chemical characteristics of milk from Kashmiri cattle. Such properties may lead to the development of certain niche products and thereby help in the conservation of this unique germplasm which has been diluted through extensive cross breeding programmes. The results provide a significant advance in our knowledge of Kashmiri cow lactating mammary gland gene expression and valuable information for future studies and breed improvement.

## Supporting information

S1 Table Genes and primer sequences for purity check of isolated mammary epithelial cells (a). Genes and primer sequences used for validation of RNA-Seq data by qPCR (b).
(DOCX)

S2 Table Read mapping statistics. (DOCX)

S3 Table Expression levels (FPKM values) of genes in mammary epithelial cells of Kashmiri and Jersey cattle^1^ at three different stages of lactation (a); expressed genes at different stages of lactation (D15, D90 and D250) in (b) Kashmiri and (c) Jersey cattle. (XLSX)

S4 Table Differentially expressed genes between (a) D15 vs D90, (b) D15 vs D250 and (c) D90 vs D250 in Kashmiri cattle; and (d) D15 vs D90, (e) D15 vs D250 and (f) D90 vs D250 in Jersey cattle; and (g) D15 vs D15, (h) D90 vs D90 and (i) D250 vs D250 between Kashmiri and Jersey cattle. (XLSX)

S5 Table Protein-Protein interaction between top 20 differentially expressed major milk candidate genes at each lactation stage comparison in (a) Kashmiri and (b) Jersey cattle using STRING database. (XLSX)

S6 Table Enriched gene ontology (GO) terms by differentially expressed genes in (a) Kashmiri cattle and (b) Jersey cattle using GO consortium database (PANTHER). (XLSX)

S7 Table Enriched pathways by differentially expressed genes in (a) Kashmiri cattle and (b) Jersey cattle using GO consortium database (PANTHER). (XLSX)
