# Supplementary material for: Comparative transcriptome analysis of mammary epithelial cells at different stages of lactation reveals wide differences in gene expression and pathways regulating milk synthesis between Jersey and Kashmiri cattle
Source: PLoS One. 2019 Feb 5;14(2):e0211773. doi: 10.1371/journal.pone.0211773 (PMC6363229; doi:10.1371/journal.pone.0211773)
Supplement: S1 Table — Genes and primer sequences for purity check of isolated mammary epithelial cells (a). Genes and primer sequences used for validation of RNA-Seq data by qPCR (b). (DOCX) [file pone.0211773.s001.docx]

**Table S1a:** Genes and primer sequences used for purity check of isolated mammary epithelial cells by qPCR

| **Gene** | **Primer sequence** | **Annealing temperature** | **Product size** |
| --- | --- | --- | --- |
| *GAPDH* | Forward-GCAAGTTCCACGGCACAG | 60°C | 213 bp |
|  | Reverse-GGTTCACGCCCATCACAA |  |  |
| *UXT* | Forward- TGGACCATCGTGACAAGGTA | 60°C | 155bp |
|  | Reverse- TGAAGTGTCTGGGACCACTG |  |  |
| *CSN2* | Forward- CAGAAAGCAGTGCCCTATCC | 60°C | 169bp |
|  | Reverse- GCCATATTTCCAAGTCGCAGT |  |  |
| *KRT-18* | Forward- GGAGAGCAAAATCCGGGAAC | 61°C | 122 bp |
|  | Reverse-GGGCGTTGTCCACAGAATTT |  |  |
| *LSP1* | Forward- CTCCAACATGGCACTCACTG | 60°C | 160 bp |
|  | Reverse- GTGGCTCTTGACCTGGAGAC |  |  |
| *HBA* | Forward- TGCCCTGTCTGAACTGAGTG | 60°C | 169 bp |
|  | Reverse- CGGTATTTGGAGGTCAGCAC |  |  |
| *CD18* | Forward- CAAACTGGCAGAAAGCAACA | 60°C | 183 bp |
|  | Reverse- TCCAGGAAGACTCTGGAGGA |  |  |

**Table S1b: Gene and primer sequences used for validation of RNA-Seq data by qPCR**

| **Gene** | **Primer sequence** | **Annealing temperature** | **Product size** |
| --- | --- | --- | --- |
| *GAPDH* | Forward-GCAAGTTCCACGGCACAG | 60°C | 213 bp |
|  | Reverse -GGTTCACGCCCATCACAA |  |  |
| *UXT* | Forward- TGGACCATCGTGACAAGGTA | 60°C | 155bp |
|  | Reverse- TGAAGTGTCTGGGACCACTG |  |  |
| *GPAM* | Forward -GGCTTCCAGATACCACTGTTT | 59°C | 199 bp |
|  | Reverse - TGGGTCCTTTTGTTTGCTTT |  |  |
| *BDH1* | Forward - CAACAAGATGTGGGAGGA | 57°C | 189 bp |
|  | Reverse - GGTAGCGAGTGTAGGGAGT |  |  |
| *SLC2A1* | Forward - GGTTTTGCCTATTCCCGTCT | 60°C | 157 bp |
|  | Reverse - GTGATCATCTGGCCCTCAGT |  |  |
| *SLC2A8* | Forward - AGGCATCCTCCTAGCCTACC | 60°C | 168 bp |
|  | Reverse - GGCATGAAACACATGAGCAG |  |  |
| *HK2* | Forward - GAGGAGAGGGGTGAGTAGGG | 60°C | 191 bp |
|  | Reverse - TCACACGCAGTGTTCACGTA |  |  |
| FAS | Forward - CTGAGTCGGAGAACCTGGAG | 60°C | 232 bp |
|  | Reverse - ACAATGGCCTCGTAGGTGAC |  |  |
| SOS2 | Forward - GCTGAAACGGAGCTTGAATC | 60°C | 153 bp |
|  | Reverse - GAGGACTGGAGCAAAGATGC |  |  |
| XDH | Forward - ACTAACACGGTGCCCAACTC | 60°C | 221 bp |
|  | Reverse - CTGTAGCCAAGGTTGGGTGT |  |  |
|  |  |  |  |
