# Supplementary material for: Comparative transcriptome analysis of mammary epithelial cells at different stages of lactation reveals wide differences in gene expression and pathways regulating milk synthesis between Jersey and Kashmiri cattle
Source: PLoS One. 2019 Feb 5;14(2):e0211773. doi: 10.1371/journal.pone.0211773 (PMC6363229; doi:10.1371/journal.pone.0211773)
Supplement: S2 Table — (DOCX) [file pone.0211773.s002.docx]

| **Sample** | **Total**  **Reads** | **QC Passed** | **QC**  **Passed %** | **Aligned**  **Read Count** | **Aligned %** | **Unaligned**  **Read Count** | **Unaligned %** |
| --- | --- | --- | --- | --- | --- | --- | --- |
| **CB-390** | 96,249,540 | 94,744,250 | 98.44 | 85,869,238 | 90.63 | 8,875,012 | 9.37 |
| **CB-390-2** | 92,517,372 | 91,969,506 | 99.41 | 84,113,665 | 91.46 | 7,855,841 | 8.54 |
| **CB-390-3-** | 116,401,530 | 115,758,07 | 99.45 | 106,605,825 | 92.09 | 9,152,253 | 7.91 |
| **CB-447** | 91,214,046 | 89,570,316 | 98.20 | 79,097,456 | 88.31 | 10,472,860 | 11.69 |
| **CB-477-2** | 93,235,128 | 89,955,200 | 96.48 | 81,828,632 | 90.97 | 8,126,568 | 9.03 |
| **CB-477-3-** | 136,828,434 | 135,768,664 | 99.23 | 125,418,364 | 92.38 | 10,350,300 | 7.62 |
| **CB-90** | 90,605,106 | 76,773,936 | 84.73 | 69,093,481 | 90.00 | 7,680,455 | 10.00 |
| **CB-90-3-** | 77,649,922 | 70,905,710 | 91.31 | 65,212,246 | 91.97 | 5,693,464 | 8.03 |
| **MG-BL1** | 82,462,774 | 72,319,896 | 87.70 | 62,308,532 | 86.16 | 10,011,364 | 13.84 |
| **MG-BL1-2** | 73,887,336 | 73,339,212 | 99.26 | 67,574,186 | 92.14 | 5,765,026 | 7.86 |
| **MGBL-1-3** | 92,098,428 | 89,792,054 | 97.49 | 84,924,806 | 94.58 | 4,867,248 | 5.42 |
| **MG-ML2** | 81,811,960 | 71,855,586 | 87.83 | 59,079,820 | 82.22 | 12,775,766 | 17.78 |
| **MG-ML2-2** | 68,425,836 | 66,722,732 | 97.51 | 59,373,810 | 88.99 | 7,348,922 | 11.01 |
| **MGML-2-3** | 87,720,824 | 86,711,472 | 98.85 | 80,073,868 | 92.35 | 6,637,604 | 7.65 |
| **MG-SL3** | 104,571,152 | 102,595,298 | 98.11 | 90,594,073 | 88.30 | 12,001,225 | 11.70 |
| **MG-SL3-2** | 76,974,734 | 76,313,042 | 99.14 | 70,145,228 | 91.92 | 6,167,814 | 8.08 |
| **MGSL-3-3** | 120,888,074 | 113,697,196 | 94.05 | 105,425,162 | 92.72 | 8,272,034 | 7.28 |
| **MG-SL3** | 68,425,736 | 66,722,732 | 97.51 | 59,293,810 | 88.23 | 7,458,922 | 10.01 |
| **Total or average** | 1,651,967,932 | 1,469,756,802 | 95.82 | 1,436,032,202 | 90.30 | 149,512,678 | 9.60 |

**S2 Table**: Read mapping statistics
